# Sugar Derivatives of Morphine: A New Window for the Development of Potent Anesthetic Drugs

**DOI:** 10.1007/s13659-015-0060-8

**Published:** 2015-05-22

**Authors:** Shyamal K. Jash, Dilip Gorai

**Affiliations:** Department of Chemistry, Saldiha College (Affiliated to the University of Burdwan), Saldiha, Bankura, 722 173 West Bengal India; Department of Chemistry, Kulti College (Affiliated to the University of Burdwan), Kulti, Burdwan, 713 343 West Bengal India

**Keywords:** Morphine, Morphine glycosides, Synthesis, Bioavailability, Anesthetic agents, Related patents

## Abstract

This review provides a short account of carbohydrate derivatives of an important natural drug, morphine, along with their comparative efficacies as anesthetic agent. Sugar derivatives are found to have more prospect as anesthetic drug than morphine itself owing to their enhanced bioavailability. Synthetic schemes of these sugar derivatives and information on related patents are also included in this manuscript.

## Introduction

Morphine (**1**) is a natural product occurring in the opium poppy *Papaver somniferous* and was first isolated in 1803 by the German pharmacist Friedrich Wilhelm Sertürner, who named it “morphium,” derived from “Morpheus”—the Greek God of Dreams [1]. This natural product has been used as the most important pain killer over many decades (or centuries as opium) and it remains the most useful choice for treatment of moderate to severe pain, either acute or chronic [2, 3]. The pharmacological properties observed for morphine, including analgesia, respiratory depression, and inhibition of gastrointestinal transit, are mediated by opioid receptors [4]. However, its use as analgesic drug has not been so much explored owing to its limited bioavailability as well as its pronounced toxicity. Therefore, it is of huge urge of scientific communities for searching morphine derivatives having greater bioavailability and of low toxicity so as to develop promising analgesic drugs. Thus structural modification (semi-synthesis) of morphine (**1**) is very much significant in this context.

Glycosylation of natural products in some cases is considered as an effective structural modification responsible for enhancing hydrophilicity of the molecules concerned—as a result of which pharmacokinetic and/or pharmacodynamic properties may be improved, although literature survey reveals that drugs having sugar moiety have generally less hydrophilicity compared to its non-sugar analogue. In some cases, attachment of a glycosyl residue provides a new chemical entity (prodrug), which facilitates the drug delivery in a more effective manner. It has been found that inclusion of carbohydrate moieties in a drug may increase its bioavailability and decrease toxicity. Therefore, sugar derivative of a drug may be more effective in treating a disease in such cases. We have attempted in this manuscript to focus some way-outs for searching new promising anesthetic drugs having morphine skeleton by considering different carbohydrate derivatives of morphine along with their comparative efficacies as anesthetic agent mentioning also the synthetic schemes used for their synthesis.

## Synthesis of Morphine Sugar Derivatives

**Fig. 1:**
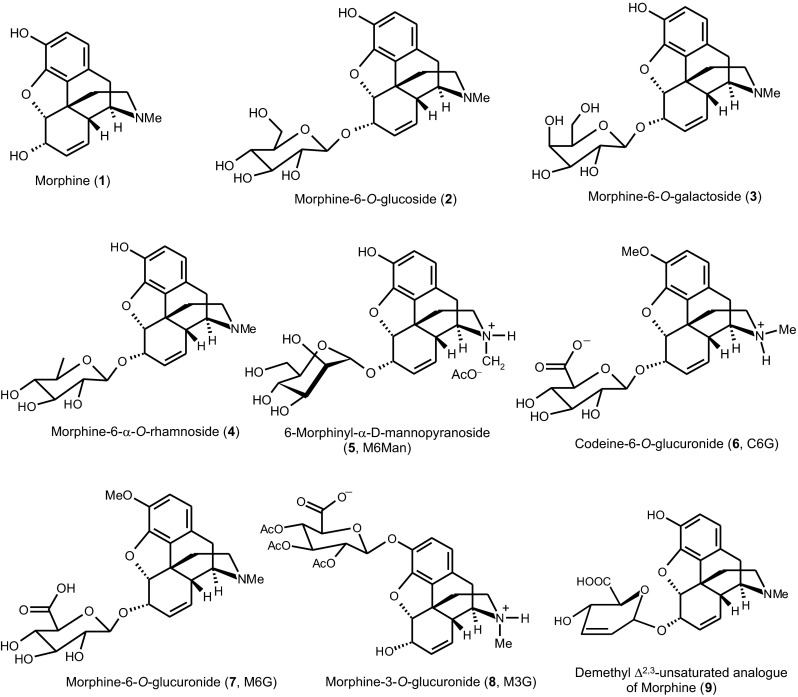

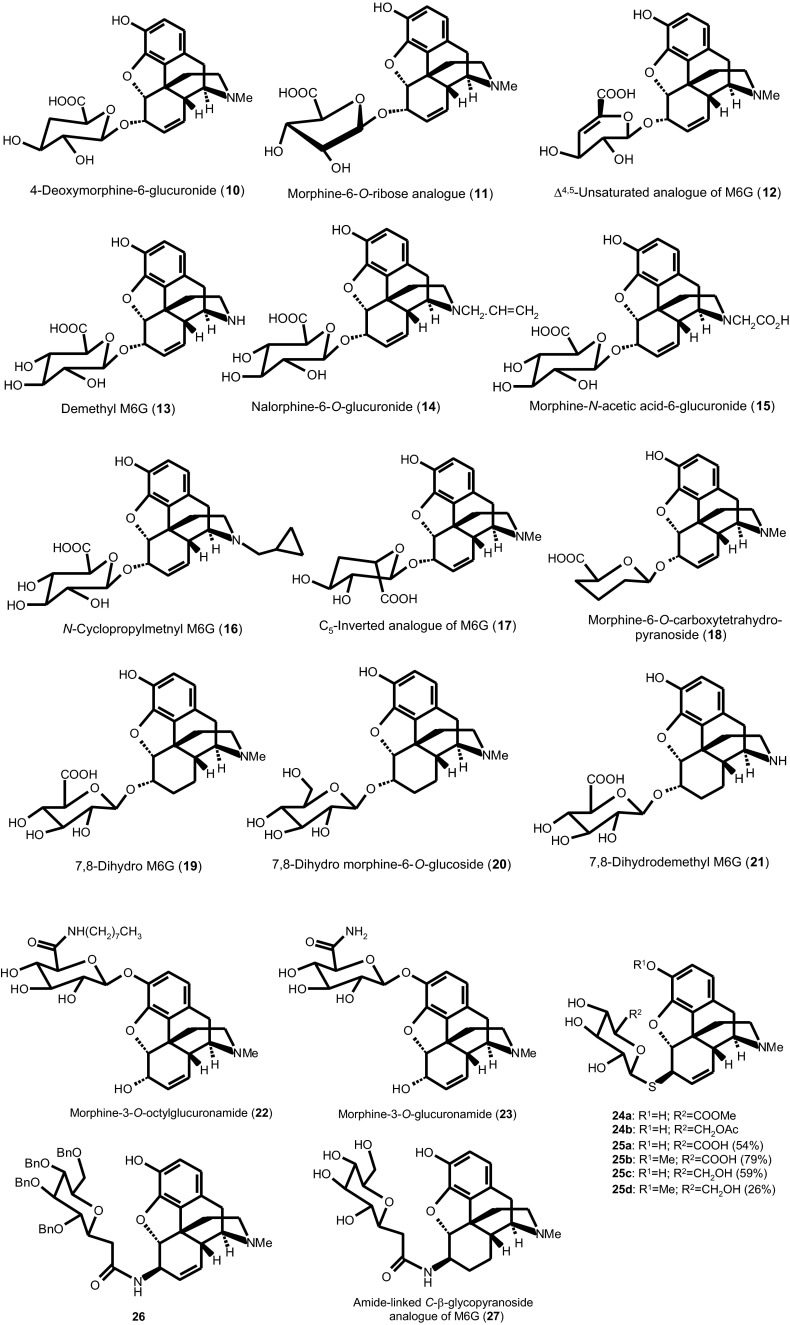
Morphine (**1**) and its sugar derivatives

In order to search better anesthetic agent, many sugar derivatives of morphine (**1**) have been synthesized (Fig. 1). In the following Table 1 a notable number of such sugar derivatives have been presented and their synthetic schemes have been discussed later on. A comparative discussion of sugar derivatives as anesthetic agents has been presented in the bioactivity section. Sugar derivatives of morphine are presented in the following Table 1 and corresponding synthetic schemes are given.

**Table 1:** Sugar derivatives of morphine (**1**)

Sl. no.	Sugar derivatives of morphine (str. no.)	Scheme no.	References
1	Morphine-6-*O*-glucoside (**2**)	Scheme 1	[5, 6]
2	Morphine-6-*O*-galactoside (**3**)	Scheme 1	[5, 6]
3	Morphine-6-α-*O*-rhamnoside (**4**)	Scheme 2	[6]
4	6-Morphinyl-α-*O*-mannopyranoside (**5**, M6Man)	Scheme 3	[9]
5	Codeine-6-*O*-glucuronide (**6**, C6G)	Scheme 4	[12]
6	Morphine-6-*O*-glucuronide (**7**, M6G)	Scheme 5	[12]
7	Morphine-3-*O*-glucuronide (**8**, M3G)	Scheme 5	[12]
8	Demethyl Δ^2,3^-unsaturated analogue of morphine (**9**)	Scheme 2	[6]
9	4-Deoxymorphine-6-glucuronide (**10**)	Scheme 6	[6]
10	Morphine-6-*O*-ribose analogue (**11**)	Scheme 7	[6]
11	Δ^4,5^-Unsaturated analogue of M6G (**12**)	Scheme 8	[6]
12	Demethyl M6G (**13**)	Scheme 8	[6]
13	Nalorphine-6-*O*-glucuronide (**14**)	Scheme 8	[6]
14	Morphine-*N*-acetic acid-6-glucuronide (**15**)	Scheme 8	[6]
15	*N*-cyclopropylmethyl M6G (**16**)	Scheme 8	[6]
16	C5-Inverted analogue of M6G (**17**)	Scheme 9	[6]
17	Morphine-6-*O*-carboxytetrahydropyranoside (**18**)	Scheme 9	[6]
18	7,8-Dihydro M6G (**19**)	Scheme 10	[6]
19	7,8-Dihydro morphine-6-*O*-glucoside (**20**)	Scheme 10	[6]
20	7,8-Dihydrodemethyl M6G (**21**)	Scheme 10	[6]
21	Morphine-3-*O*-octylglucuronamide (**22**)	Scheme 11	[17]
22	Morphine-3-*O*-glucuronamide (**23**)	Scheme 11	[17]
23	Morphine-6-*S*-glucuronide (**24a**)	Scheme 12	[4]
24	Codeine-6-*S*-glucuronide (**24b**)	Scheme 12	[4]
25	Morphine-6-*S*-glucoside (**24c**)	Scheme 12	[4]
26	Codeine-6-*S*-glucoside (**24d**)	Scheme 12	[4]
27	Amide-linked *C*-β-glycopyranoside analogue of M6G (**27**)	Scheme 13	[19]

**Scheme 1:**
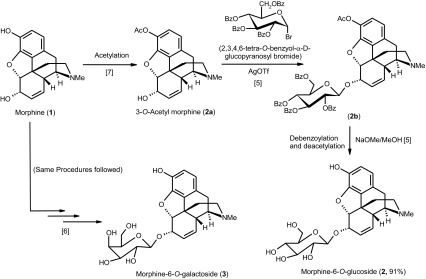
Synthesis of morphine-6-*O*-glucoside (**2**) and morphine-6-*O*-galactoside (**3**) [5, 6]

In 1995, Kovac and Rice [5] first reported the synthesis of morphine-6-*O*-glucoside (**2**) from 3-*O*-acetylmorphine (**2a**). The synthesis involves glucosilation to form a glycoside followed by debenzoylation and deacetylation. Highest yields of glycoside **2** (about 91 %) were obtained when 2,3,4,6-tetra-*O*-benzoyl-α-d-glucopyranosyl bromide is used a glycosyl donor, and condensation was promoted with silver triflate. Following the same procedures another sugar derivative, morphine-6-*O*-galactoside (**3**) was prepared (Scheme 1) [5, 6].

**Scheme 2:**
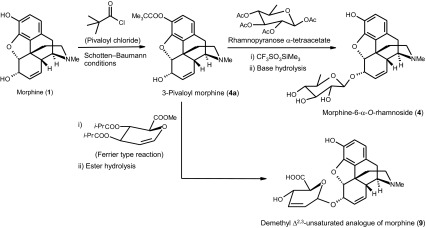
Synthesis of morphine-6-α-*O*-rhamnoside (**3**) and demethyl Δ^2,3^-unsaturated analogue of morphine (**9**) [6]

The morphine-6-α-*O*-rhamnoside (**4**) was synthesized by direct coupling of rhamnopyranose α-tetraacetate to 3-pivaloyl morphine (**4a**) by CF_3_SO_3_SiMe_3_ followed by base hydrolysis of the esters (Scheme 2) [6]. The compound **4a** was prepared by two phase acylation of morphine with pivaloyl chloride under Schotten–Baumann conditions [7, 8]. The demethyl Δ^2,3^-unsaturated analogue of morphine (**9**) was prepared by a Ferrier type reaction of 3-pivaloylmorphine (**4a**) with glycal, followed by ester hydrolysis (Scheme 2) [6].

**Scheme 3:**
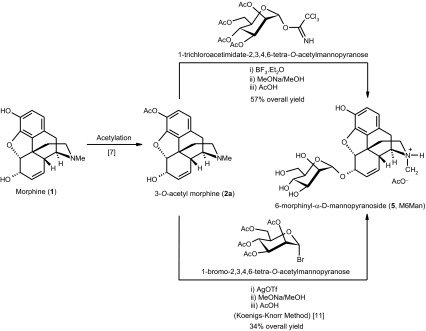
Synthesis of 6-methyl-α-d-mannopyranoside (**5**, M6Man) [9]

6-Morphinyl-α-*O*-mannopyranoside (**5**, M6Man) was synthesized by Arsequell et al. [9] starting from 3-*O*-acetylmorphine (**2a**) applying two procedures. In first procedures 3-*O*-acetylmorphine (**2a**) [10] and 1-trichloroacetimidate-2,3,4,6-tetra-*O*-acetyl-mannopyranose are mixed and stirred under an argon atmosphere at 0 °C until the addition of BF_3_·Et_2_O complex. After 22 h of reaction at room temperature, the mixture was diluted with methylene chloride, washed with sodium bicarbonate, purified, and deacetylated, yielding the 6-morphinyl-α-*O*-mannopyranoside (**5**) in a 57 % overall yield (Scheme 3). In the second method (Koenigs–Knorr Method [11]) the mannoside (**5**) was obtained in 34 % overall yield (Scheme 3) [9].

**Scheme 4:**
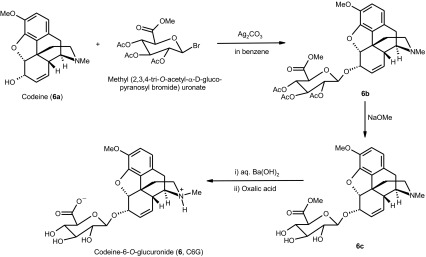
Synthesis of codeine-6-*O*-glucuronide (**6**, C6G) [12]

In 1968 Yoshimura et al. [12] developed a concise method to prepare codeine-6-*O*-glucuronide (**6**, C6G) from codeine (**6a**) by utilization of Koenigs–Knorr reaction [11] (Scheme 4). Morphine-6-*O*-glucuronide (**7**, M6G) was also synthesized similarly to that of compound **6** utilizing 3-*O*-acetylmorphine (**2a**) as the starting material which was prepared quantitatively by selective acetylation of morphine according to the method of Welsh [10].

**Scheme 5:**
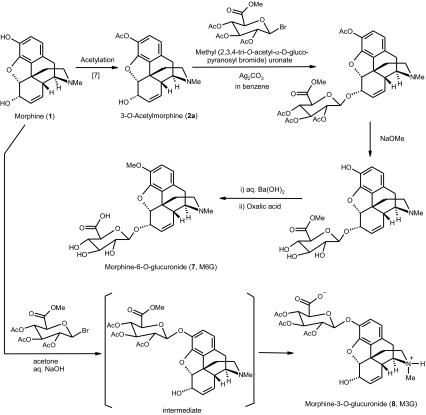
Synthesis of morphine-6-*O*-glucuronide (**7**, M6G) and morphine-3-*O*-glucuronide (**8**, M3G) [12]

The highly active compound **7** was obtained from morphine (**1**) by means of a series of reactions as depicted in Scheme 5 [12]. Similarly synthesis of morphine-3-*O*-glucuronide (**8**, M3G) was performed by the employment of sodium hydroxide as the condensing agent in aqueous acetone solution (Scheme 5). The method used was essentially the same as those by Mannich [13] and by Casparis and Bechert [14].

**Scheme 6:**
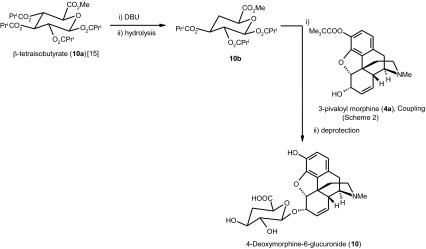
Synthesis of 4-deoxymorphine-6-glucuronide (**10**) [6]

**Scheme 7:**
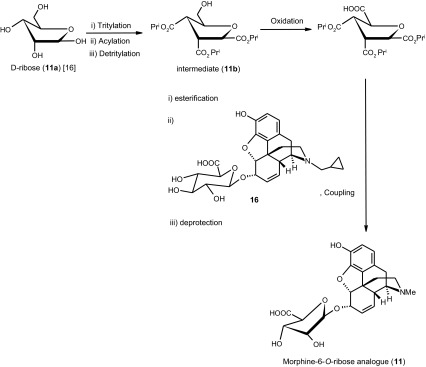
Synthesis of morphine-6-*O*-ribose analogue (**11**) [6]

4-Deoxymorphine-6-glucuronide (**10**) was obtained from β-tetraisobutyrate (**10a**) [15] by a sequence of elimination using DBU followed by hydrogenation; the major 5β-isomer (**10b**) was crystallised and was coupled to **4a** followed by deprotection to give 4-deoxymorphine-6-glucuronide (**10**) (Scheme 6) [6]. Morphine-6-*O*-ribose analogue (**11**) was prepared via a sequence of tritylation of d-ribose (**11a**) [16] followed by acylation and detritylation to give intermediate **11b**. Following oxidation to the 5-carboxylic acid and esterification, the fully protected sugar was coupled as an α/β-mixture to *N*-cyclopropyl methyl M6G (**16)**; subsequent deprotection gave morphine-6-*O*-ribose analogue (**11**) as a single (β-) anomer (Scheme 7) [6].

**Scheme 8:**
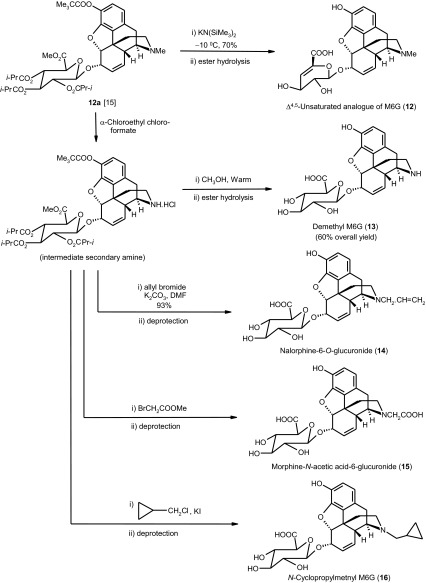
Synthesis of Δ^4,5^-unsaturated analogue of M6G (**12**), demethyl M6G (**13**), Nalorphine-6-*O*-glucuronide (**14**), Morphine-*N*-acetic acid-6-glucuronide (**15**) and *N*-cyclopropylmethyl M6G (**16**) [6]

**Scheme 9:**
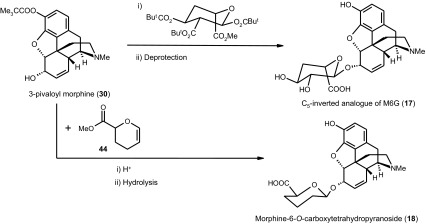
Synthesis of C_5_-inverted analogue of M6G (**17**) and Morphine-6-*O*-carboxytetrahydropyranoside (**18**) [6]

**Scheme 10:**
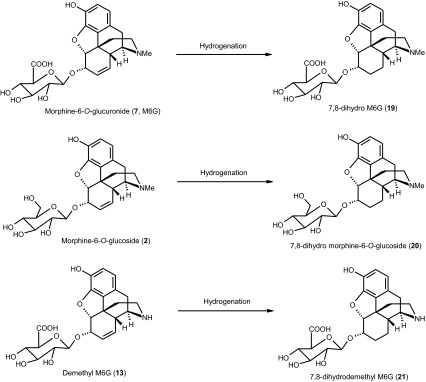
Synthesis of 7,8-dihydro M6G (**19**), 7,8-dihydro morphine-6-*O*-glucoside (**20**) and 7,8-dihydrodemethyl M6G (**21**) [6]

Stachulski and his group [6] accomplished the synthesis of various types of analogues of morphine (**1**). The starting compound **12a** [15] underwent many series of reactions to furnish Δ^4,5^-unsaturated analogues of M6G (**12**), demethyl M6G (**13**), nalorphine-6-*O*-glucuronide (**14**), morphine-*N*-acetic acid-6-glucuronide (**15**) and *N*-cyclopropylmethyl M6G (**16**) as depicted in Scheme 8 [6]. The same research group [6] also accomplished the synthesis of C5-inverted analogue of M6G (**17**) and morphine-6-*O*-carboxytetrahydropyranoside (**18**) from 3-pivaloyl morphine **4a** (Scheme 9). And also 7,8-dihydro analogues (**19**–**21)** of M6G, morphine-6-*O*-glucoside and demethyl M6G were obtained by hydrogenation of **7**, **2** and **13**, respectively (Scheme 10); **20** was conveniently isolated as its succinate salt, while **19** and **21** were kept in their zwitterionic forms [6].

**Scheme 11:**
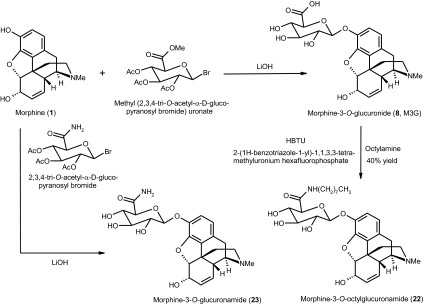
Synthesis of lipophilic M3G analogue, morphine-3-*O*-octylglucuronamide (**22**) and morphine-3-*O*-glucuronamide (**23**) [17]

Salvatella and his group [17] have synthesized a lipophilic M3G analogue, morphine-3-*O*-octylglucuronamide (**22**) (Scheme 11). The simplest route was to form an amide bond between the carboxylic acid group on M3G (**8**) and a primary linear alkyl amine. Accordingly, the whole procedure for morphine-3-*O*-octylglucuronamide (**22**) synthesis started following a one-pot reaction method to prepare M3G (**8**). This glycosidation reaction departed from morphine (**1**) and methyl (2,3,4-tri-*O*-acetyl-α-d-glucopyranosyl bromide) uronate as substrates and used LiOH as promoter and base for the removal of acetyl protecting groups [18]. In a second step, the coupling reaction between the glucuronide M3G (**8**) and octylamine was accomplished by means of the uronium salt HBTU [2-(1H-benzotriazole-1-yl)-1,1,3,3-tetramethyluronium hexafluoro-phosphate] afforded 40 % yield after crystallization from acetone/water (Scheme 11) [17]. Similarly, morphine-3-*O*-glucuronamide (**23**) was prepared from morphine (**1**) and the corresponding acetylated glucuronamide bromide derivative also using the LiOH method (Scheme 11) [17].

**Scheme 12:**
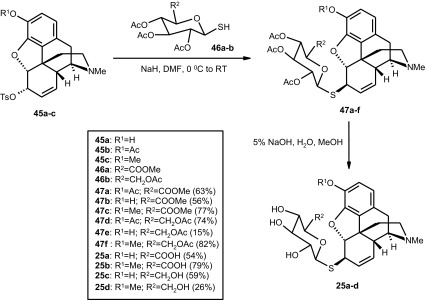
Synthesis of M6G thiosaccharide analogues (**25a**–**d**) [4]

A series of 6-β-thiosaccharide (**25a**–**d**) analogues of morphine-6-glucuronide (**7**) and codeine-6-glucuronide (**6**) were synthesized by MacDougall [4]. The starting compounds for the preparation of the thiosaccharides **47a**–**f** and **25a**–**d** were 6-*O*-tosylmorphine **45a**, 3-*O*-acetyl-6-tosylmorphine **45b**, and 3-*O*-tosylcodeine **45c** [10]. The tosylates **45a**–**c** were prepared in good yield by the reaction of either 3-*O*-acetylmorphine or codeine with *p*-toluenesulfonyl chloride in pyridine at 3 °C overnight. The key step in the synthesis of **47a**–**f** was the attachment of the thiosaccharide to the phenanthrene nucleus by an S_N_2 displacement reaction (Scheme 12) [4].

**Scheme 13:**
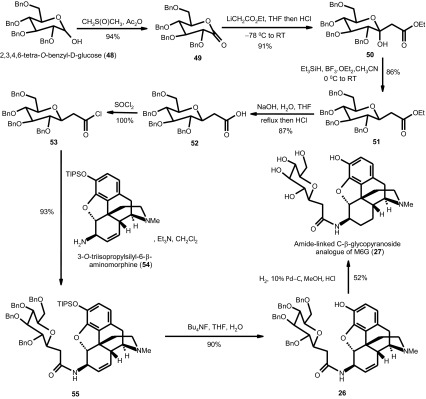
Synthesis of amide-linked *C*-β-glycopyranoside analogue of M6G (**27**) [19]

A eight-step synthesis of amide-linked *C*-β-glycopyranoside analogue of M6G (**27**) was achieved by MacDougall and his group [19] using 3-triisopropylsilyl-6-β-aminomorphine and 2,3,4,6-tetra-*O*-benzyl-d-glucose (Scheme 13).

## Biological Activities

There are three major types of opioid receptors: mu (μ), delta (δ) and kappa (κ). Morphine (**1**) is an agonist and partial agonist of μ and δ opioid receptors, respectively [20]. Opioid receptors are mainly localized in the limbic system. They are involved in the control of emotion and reward behaviors; the ascending and descending pain pathways that include the different laminae layers of cortex, thalamus, periaqueductal grey, midbrain median raphae and the dorsal horn of the spinal cord; and specific brain regions that are known to control locomotion, emesis, cough and temperature [20]. Some pharmacological effects associated with opioid receptor types are presented in Table 2.

**Table 2:** Pharmacological effects associated with opioid receptor types [20]

Sl. no.	Pharmacological effects	Opioid receptors		
		mu (μ)	Delta (δ)	Kappa (κ)
1.	Analgesia	Supraspinal	Supraspinal	−
		Spinal	Spinal	Spinal
2.	Pupil constriction	++	++	−
3.	Respiratory depression	+++	++	+
4.	Diuresis	Antidiuresis	−	+
5.	Gastrointestinal	Constipation	Constipation	−
6.	Smooth muscle	Spasm	Spasm	−
7.	Behavior/affect	Euphoria	−	Dysphoria
		Sedation	−	Sedation
8.	Physical dependence	++	++	+

(−) no effect; (**+**) low effect; (**++**) moderate effect; (**+++**) high effect

In human beings, morphine (**1**) is predominantly metabolized by hepatic glucuronosyl transferases with the addition of glucuronic acid at 3-*O*-position in the aromatic ring or at 6-*O* position on the phenanthrene ring, furnishing the morphine 3-*O*-glucuronide (**8**, M3G) and morphine 6-*O*-glucuronide (**7**, M6G), respectively [21, 22]. Approximately 10 % of morphine (**1**) is metabolized to M6G (**7**) and 50 % to M3G (**8**) [23]. Many reports have disclosed the very low affinity of **8** to μ-opioid receptors [24] and, in a small study in humans, it appeared to be devoid of significant analgesic activity [25]. However, the analgesic potency of **7** in animals with significantly reduced respiratory depression, nausea, and sedation is about 100 times greater than that of morphine [26, 27]. It has been found that the high potency of M6G (**7**) as an analgesic is mediated through opioid receptors [28]. These observations are apparently controversial due to the polar properties of **7**. Villesen et al. [29] studied pharmacokinetics of M6G (**7**) on healthy volunteers and proposed that M6G is hydrolysed to morphine **1** in the colon, which is then absorbed and subsequently undergoes metabolism in the liver to morphine-3-glucuronide (**8**, M3G) and M6G [29]. M6G (**7**) is also able to penetrate the blood–brain barrier, although this occurs in a lower extent when compared to morphine [30–32]. Two possible mechanisms for this phenomenon have been proposed. The first one lies on the active transport of M6G across blood–brain barrier through a glucose transporter. There is an evidence on modulation of M6G entry into brain by membrane P-glycoprotein (Pgp) [33–35]. The second hypothesis is based on partitioning experiments and computer simulations and suggests that M6G molecules act like “molecular chameleons” by adopting a confirmation of lower polarity when passing the blood-brain barrier (BBB) [36–38]. Research is going on only to include sugar moiety at 6-position and to compare their anesthetic potential with morphine in order to develop new improved anesthetic drug. Therefore, limited reports are expected. In summary, the reduction in side-effects and enhanced potency observed for M6G make this morphine glycoderivative a promising drug candidate for the treatment of cancer-related pain as well as a lead compound for further development of new drugs.

Recently, non-glucuronic analogues (**2**–**4** and **10**–**11**) of M6G were synthesized. Affinities of these morphine glycosides to μ receptor as well as in vivo antinociceptive activities using the hotplate method were also described [6]. This study shows that β-glucoside **2** was the only compound with significant antinociceptive activity at doses of 2 and 4 mg/kg, along with slightly higher affinity for μ_1_ receptor. Then, the constant of receptor inhibition (*k*i) for compound **2** was 0.28 nM whereas morphine (**1**) or M6G (**7**) presented a *k*i of 0.78 and 1.5 nM, respectively. When rhamnoside substitutes glucose (analogue **4**) the *k*i for this morphine glycoderivative towards μ_1_ receptor becomes 0.17 nM. However, its antinociceptive activity is very low. The galactoside **3** (*k*i = 1.2 nM) had similar μ_1_ affinity to morphine (**1**) and M6G (**7**), showing some antinociceptive activity at 4 mg/kg and significant one at 8 mg/kg. All glycoderivatives studied (**2**–**4**) presented affinity to μ_2_ receptor similar to that for morphine. The analogues **10** and **11**, which possess acid sugar residues, showed slightly higher μ_1_ affinity, but reduced antinociception when compared with morphine (**1**) [6]. In general, Stachulski and coworkers disclosed that modification of the carbohydrate at 6-*O*-position of morphine promotes marked in vitro effects on binding to μ opioid receptor subtypes μ_1_ or μ_2_ and in vivo antinociceptive activity [6]. 6-morphinyl-α-d-mannopyranoside (**5**) is found to have 100-fold higher naloxone-reversible antinociception activity and twice as long lasting compared to morphine (**1**) when administered intraperitoneally to rats. Moreover, this compound **5** does not produce tolerance and binds to rat *μ* opioid receptors with two fold affinities than morphine **1**. It has been concluded on the basis of NMR studies that differences of activity between the derivative and its parent compound M6G (**7**) might be related to their differing molecular dynamic behavior [9].

Although M6G (**7**) and some of its *O*-glycosides present significantly greater analgesic potency than morphine (**1**), the bioavailability of these compounds could be a problem for a useful drug. For instance, the oral bioavailability of M6G is only 11 % and improvement of chemical and metabolic stability of M6G could possibly increase its effectiveness as a potential drug. Limited hydrophilicity has been a major problem in most of the cases for morphine sugar derivatives making obstacles to be used in clinic. However, examples are known which are in Clinical trials e.g. M6G is being developed by CeNeS (Cambridge, UK) as a treatment for postoperative pain, and is currently undergoing phase III trials in Europe, with phase III clinical trials in the USA expected to commence in 2007 [39]. A general strategy for improving in vivo metabolic stability of glycoconjugates involves the replacement of glycosidic oxygen atom by carbon, nitrogen, or sulfur atoms. Indeed, MacDougall and coworkers have developed a new series of sulfur (**24a, 24b, 25a and 25c**) and carbon (**26** and **27**) glycosides (Fig. 1). The results from this research indicate some compounds with metabolic stability for sustained pharmacological activity [4, 19]. Compounds **24a** and **24b** were full μ receptor agonists, whereas compounds **25a** and **25c** were only partial agonists. This finding indicates that the presence of a hydrogen atom (as a hydrogen bound donor) or a carboxyl group at C-6′ in sugar moiety is not a requirement for agonist activity of these analogues. Moreover, analogue **27** and its deprotected sugar congener **26** were both μ receptor selective [19]. Compound **27** (*k*i = 0.5 nM) presented an affinity to μ opioid receptor 26-fold higher than that observed for M6G. Selectivities of compound **27** for μ versus δ and μ versus κ receptors were tenfold and 34-fold, respectively. Higher potency was also observed for **26** (3.7-fold) in comparison with M6G. Selectivity of compound **26** for μ versus δ and μ versus κ receptors were 77-fold and 166-fold, respectively. This compound showed slightly greater potency towards μ receptor (2.5-fold) over M6G (**8**) when compared to thiosaccharides **25a** and **25c** (around 1.6-fold) [19]. In addition, compound **26** presented considerable metabolic stability when assayed hepatic microsome preparations from rat and monkey. No detectable loss of **26** was observed during 90 min of system incubation. Compound **26** was also very stable at pH 2 or pH 7.4.

## Related Patents on Morphine and its Sugar Derivatives

About sixteen patents on morphine and its sugar derivatives are available, which deal with isolation technique, purification, synthesis of various sugar derivatives and their analogues, evaluation of their anesthetic activity and other pharmaceutical potentials. Such related patent information including all necessary agenda are presented in Table 3.

**Table 3:** Related patent information on morphine and its sugar derivatives

Patent number	Filing date	Issue date	Original assignee	Title	Inventors	References
US2062324	Jul 12, 1935	Dec 1, 1936	Mallory George Elwood	Method of extraction of morphine and related derivatives	George Elwood Mallory	[40]
US2715627	May 26, 1952	Aug 16, 1955	Peoria, III., assignors to the United States of America as represented by the Secretary of Agriculture	Solvent extraction of opium alkaloids	Charles L. Mehltretter and Francis B. Weakley	[41]
EP0324212A1	Jan 12, 1988	Jul 19, 1989	Baker Cummins pharmaceuticals, inc.	Glucuronic acid derivatives of opioid antagonists	Ronald R. Tuttle, Ross Dixon and Maciej M. Smulkowski	[42]
WO1993005057A1	Sep 4, 1992	Mar 18, 1993	Irepa Inst Regional De Promoti	Method for synthesizing glucuronides of 4,5-epoxy morphinanes	Alfred Adophe Henri Mertz	[43]
WO1995016050A1	Nov 29, 1994	Jun 15, 1995	Richard Talbot Brown	An enzymatic process for making morphine-6-glucuronide or substituted morphine-6-glucuronide	Richard T. Brown, Neil E. Carter, Feodor Scheinmann and Nicholas J. Turner	[44]
US5589480	Aug 17, 1994	Dec 31, 1996	–	Topical application of opioid analgesic drugs such as morphine	George F. Elkhoury and Christoph Stein	[45]
US5593695	May 24, 1995	Jan 14, 1997	ALZA Corporation, Pamo Alto, Calif.	Morphine therapy	Sonya Merrill, Atul D. Ayer, Paul Hwang and Anthony L. Kuczynski	[46]
US5621087	Feb 4, 1994	Apr 15, 1997	Salford Ultrafine Chemicals and Research Limited, Manchester, United Kingdom	Process for making morphine-6- glucuronide or substituted morphine-6-glucuronide	Feodor Scheinmann, Keith W. Lumbard, Richard T. Brown, Stephen P. Mayalarp and Neil E. Carter	[47]
US5667805	Oct 4, 1996	Sep 16, 1997	ALZA Corporation, Pamo Alto, Calif.	Morphine therapy	Sonya Merrill, Atul D. Ayer, Paul Hwang and Anthony L. Kuczynski	[48]
US5866143	Oct 15, 1996	Feb 2, 1999	El Khoury and Stein, Ltd., Long Beach, Calif.	Topical application of opioid drugs such as morphine for relief of itching and skin disease	George F. Elkhoury	[49]
US5977326	Apr 14, 1997	Nov 2, 1999	Salford Ultrafine Chemicals and Research Limited, Manchester, United Kingdom	Process for making morphine-6-glucuronide or substituted morphine-6-glucuronide	Feodor Scheinmann, Simon Joel and Andrew V. Stachulski	[50]
US6046313	Jul 31, 1996	Apr 4, 2000	Salford Ultrafine Chemicals and Research Limited, Manchester, United Kingdom	Process for making morphine-6-glucuronide or substituted morphine-6-glucuronide	Feodor Scheinmann, Keith W. Lumbard, Richard T. Brown, Stephen P. Mayalarp and Neil E. Carter	[51]
US6054584	Nov 19, 1996	Apr 25, 2000	The Board of Regents of the University and Community College, System of Neveda, Reno, Nev.	Process for extracting and purifying morphine from opium	Junning Ma and Robert, C. Corcoran	[52]
US6566510B1	Jun 4, 1999	May 20, 2003	Genes Limited, Cambridge (GB)	Morphine-6-glucuronide synthesis	Parsons, P. J. and Ewin, R. A.	[53]
EP1412368B1	Jul 24, 2002	Apr 28, 2004	Euro-Celtique S.A., 2330 Luxembourg (LU)	Sugar derivatives of hydromorphone, dihydromorphine and dihydroiso-morphine, compositions thereof and uses for treating or preventing pain	Feng Gao and Jahanara Miotto	[54]
US6740641 B2	Jul 22, 2002	May 25, 2004	Euro-Celtique S.A. (LU)	Sugar derivatives of hydromorphone, dihydromorphine and dihydroiso-morphine, compositions thereof and uses for treating or preventing pain	Feng Gao and Jahanara Miotto	[55]

## Conclusion

It may concluded from the above discussions that glycosylation of the natural product, morphine (an anesthetic drug) is responsible for the effective structural modification for enhancing hydrophilicity of the compound—as a result of which pharmacokinetic and/or pharmacodynamic properties are improved. In some cases, attachment of glycosyl residue provides a new chemical entity (prodrug), which facilitates the drug delivery in a more effective manner. A significant number of related patents have also been filed so far. Therefore, this review will be very much helpful for the direction of searching new effective anesthetic drug containing morphine skeleton in near future.

## Abbreviations

BBB: Blood–brain barrier
C6G: Codeine-6-*O*-glucuronide
DBU: 1,8-Diazabicyclo[5.4.0]undec-7-ene
HBTU: 2-(1*H*-benzotriazole-1-yl)-1,1,3,3-tetramethyluronium hexafluoro-phosphate
M3G: Morphine-3-*O*-glucuronide
M6G: Morphine-6-*O*-glucuronide
M6Man: 6-Morphinyl-α-*O*-mannopyranoside
NMR: Nuclear magnetic resonance
Pgp: P-glycoprotein
